# A Stable Switch From Bictegravir/Emtricitabine/Tenofovir Alafenamide (B/FTC/TAF) to Dolutegravir/Lamivudine (DTG/3TC) in the Absence of Historical Resistance Records: Results From the Switch to Dovato in Patients Suppressed on Biktarvy (SOUND) Cohort

**DOI:** 10.7759/cureus.82716

**Published:** 2025-04-21

**Authors:** Jihad Slim, Paul Bellafiore, Masara Touza, James P Fallon, Rebecca A Borsi

**Affiliations:** 1 Department of Infectious Diseases, Saint Michael's Medical Center, Newark, USA; 2 Department of Internal Medicine, Saint Michael's Medical Center, Newark, USA; 3 Department of Clinical Research, Saint Michael's Medical Center, Newark, USA; 4 Department of Pharmacology and Therapeutics, ViiV Healthcare, Durham, USA

**Keywords:** b/ftc/taf, cohort, dtg/3tc, hiv, resistance

## Abstract

Objective: This article aims to examine the safety and efficacy of switching from bictegravir/emtricitabine/tenofovir alafenamide (B/FTC/TAF) to dolutegravir and lamivudine (DTG/3TC) in the absence of prior resistance records.

Design: Switch to Dovato in patients suppressed on Biktarvy (SOUND) is an open-label, single-arm, pilot study of adult people with HIV who were virologically suppressed (HIV-1 <50 copies/mL) on B/FTC/TAF for >24 weeks, and switched to DTG/3TC in the absence of available resistance records.

Methods: The primary endpoint was the percentage of participants with HIV viral load (VL) ≥50 c/mL at week 48. Secondary endpoints at weeks 48 and 96 included the percentage of participants with HIV-VL <50 c/mL, incidence and severity of adverse events, laboratory abnormalities, change in baseline CD4 cell count, and retrospective proviral DNA resistance testing on banked baseline samples.

Results: Of the 40 individuals enrolled, 0% had VL ≥50 c/mL at week 48. No participants discontinued due to laboratory abnormalities or safety-related concerns. Three participants withdrew from the study while virologically suppressed. Among the 32 baseline samples available for retrospective proviral DNA resistance testing, six (19%) had nucleoside reverse transcriptase inhibitor resistance-associated mutations (RAMs), all with M184V. Two (6%) participants had integrase strand transfer inhibitor RAMs at baseline (S147S/G and Q148Q/R); neither conferred resistance to DTG. Nonnucleoside reverse transcriptase inhibitor and protease inhibitor RAMs were observed in eight (25%) and three (9%) participants, respectively. A significant decrease in weight was observed over the study period.

Conclusions: Results from SOUND support the efficacy and safety of switching to DTG/3TC for people living with HIV-1 who are virologically suppressed on B/FTC/TAF with unknown resistance history and may confer a weight advantage.

## Introduction

Current treatment guidelines for the treatment of HIV recommend two- or three-drug integrase strand transfer inhibitor (INSTI)-based therapy commonly prescribed at single-table regimens (STR) [1].  Potential advantages of using fewer drugs in an STR can be fewer drug-drug interactions, reduction in the risk of adverse events (AEs), polypharmacy, and a decrease in the cost of antiretroviral therapy (ART), all without compromising virologic suppression (VS) [2,3].  Previous studies have demonstrated that approved two-drug STRs are noninferior to three-drug STRs in terms of viral suppression for appropriate patients [4-7]. 

Two commonly used STRs are dolutegravir and lamivudine (DTG/3TC) and bictegravir/emtricitabine/tenofovir alafenamide (B/FTC/TAF). Potential barriers to using DTG/3TC include a patient's hepatitis B status and concerns about nucleoside reverse transcriptase inhibitor (NRTI) resistance, specifically the M184V mutation [8]. While this mutation is rare (about 1%) in people with HIV (PWH) naïve to treatment in the US, it is not uncommon to find it upon virologic failure (VF) in those on 3TC or FTC-containing regimens (as high as 80%) [9-11].

We were therefore interested in studying whether patients switching from B/FTC/TAF to DTG/3TC would maintain VS when prior resistance testing was not available.

## Materials and methods

Switch to Dovato in patients suppressed on Biktarvy (SOUND) is an open-label, single-arm, pilot study of adult PWH who were virologically suppressed (HIV-1 <50 copies/mL) on B/FTC/TAF and switched to DTG/3TC in the absence of available resistance records. Participants were enrolled from March 2021 to March 2022. The Saint Michael’s Medical Center Institutional Review Board approved the study, and all participants provided written consent before screening.

Participants >18 years of age who were VS on B/FTC/TAF for >24 weeks and had no available resistance testing results were eligible. Participants who had VF in the past were eligible, provided they were VS at enrollment according to the above protocol. Major exclusion criteria were a positive hepatitis B surface antigen, creatinine clearance <30 mL/minute/1.73 m^2^, or severe hepatic impairment (alanine aminotransferase greater than five times the upper limit of normal).

**Figure 1:**
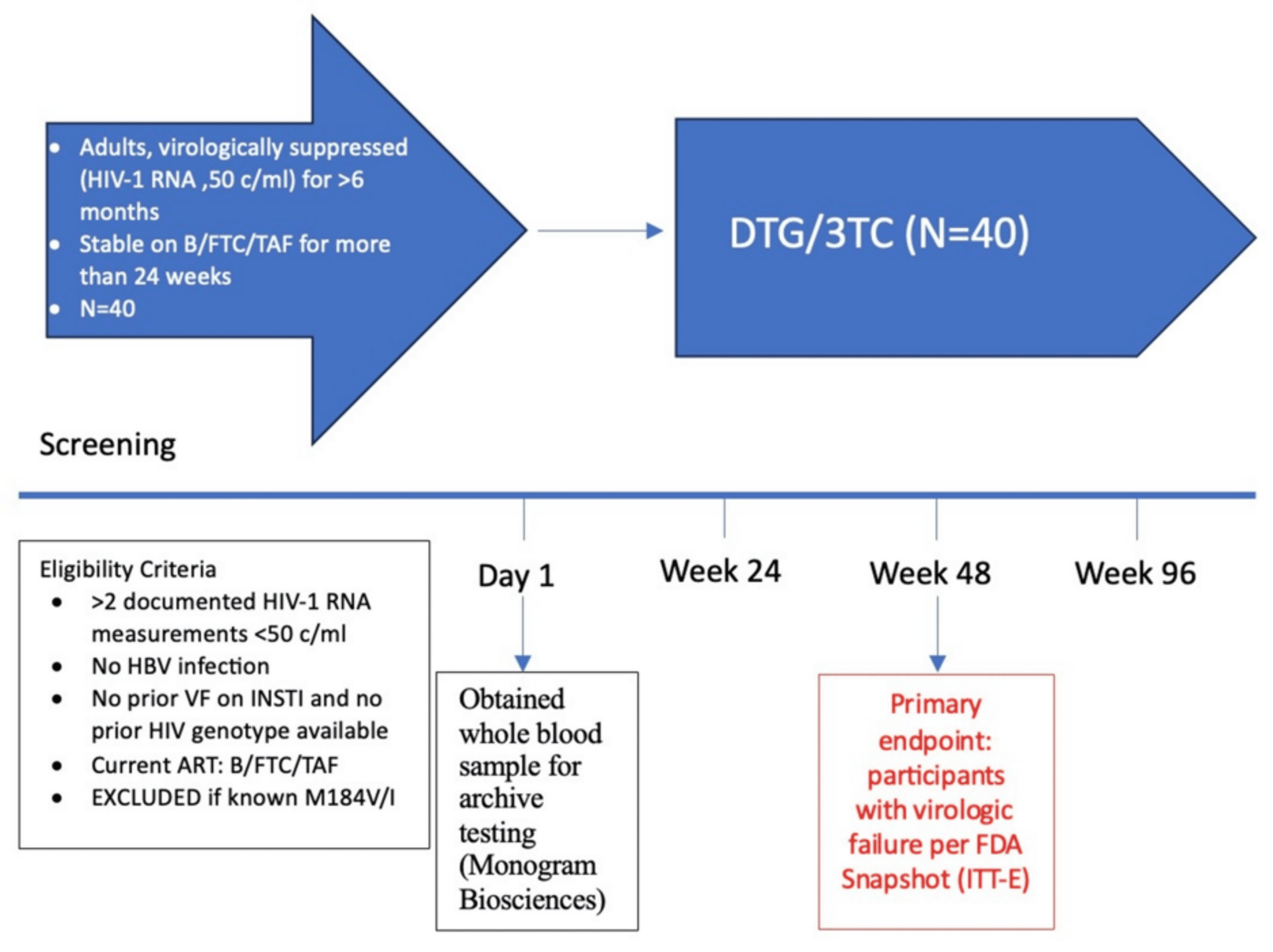
Open-label, single-center, single-arm pilot study design DTG: dolutegravir; 3TC: lamivudine; HBV: hepatitis B virus; VF: virologic failure; B/FTC/TAF: bictegravir, emtricitabine, and tenofovir alafenamide; ITT-E: intention to treat exposed; INSTI: integrase strand transfer inhibitor; ART: antiretroviral therapy

A whole blood sample for archive testing (Monogram Biosciences, South San Francisco, CA) was drawn on Day 1 before dispensing DTG/3TC to the participant (Figure 1). Participants were assessed for safety and efficacy through a physician visit and laboratory tests at weeks 4 and 12, and every 12 weeks thereafter, up to week 96. Vital signs, weight, hematology panel, chemistry panel, HIV viral load (VL), and CD4 T-cell counts were performed at every scheduled study visit.  A fasting lipid profile was performed on Day 1 and at weeks 24, 48, 72, and 96.

End points

The primary endpoint was the percentage of participants with an HIV-VL ≥50 copies/mL at week 48 using FDA intention-to-treat snapshot analysis. Secondary endpoints at weeks 48 and 96 included the percentage of participants with HIV-VL <50 copies/mL using FDA snapshot, incidence and severity of AEs, percentage of subjects who discontinued treatment due to AEs, laboratory abnormalities, change in baseline CD4 cell count, and retrospective proviral DNA testing on banked samples to compare virologic outcome for study participants (those with vs. without baseline M184I/V mutations). VF was defined as plasma HIV-1 ≥50 copies/mL confirmed by a retest plasma HIV-1 ≥200 copies/mL performed in three to four weeks.

Statistics

Participants who received at least one dose of DTG/3TC were included in the primary and secondary analyses. Basic descriptive statistics were used to analyze the demographic and baseline characteristics. AEs, significant AEs (SAEs), withdrawals, and virologic response were summarized.

## Results

Efficacy

**Figure 2:**
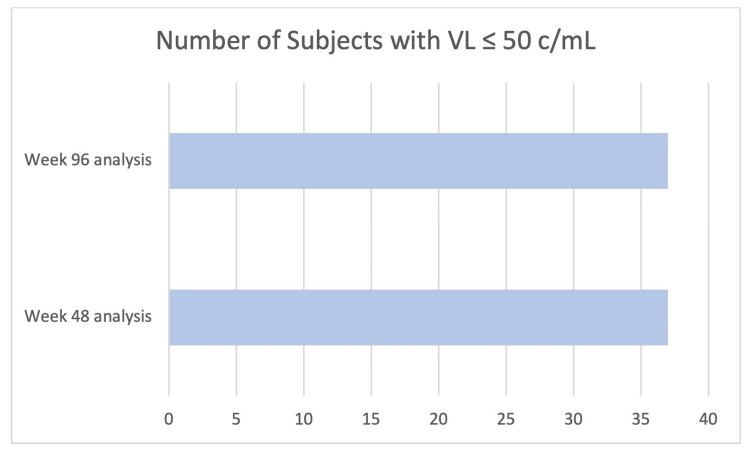
Results for secondary endpoints VL: viral load

Of the 40 individuals enrolled who switched to DTG/3TC, 0 (0%) had a VL ≥50 c/mL at week 48; 37 (93%) remained virologically suppressed (HIV-1 RNA <50 c/mL) at the same time point. Three participants withdrew through week 48 and were virologically suppressed at the time of discontinuation. One participant withdrew due to becoming pregnant, and two withdrew due to a preference for B/FTC/TAF. At week 96, all 37 participants maintained VS (HIV-1 RNA <50 c/mL), with no new discontinuations since the 48-week analysis (Figure 2).

Resistance

Forty participants had baseline samples drawn; however, 32 were available for retrospective proviral DNA resistance testing. Three patients withdrew from the study, two sample tubes cracked during transport, two samples could not be located, and one sample could not be analyzed. Six (19%) had NRTI resistance-associated mutations (RAMs), all of which harbored the M184I/V mutation. Two (6%) participants had INSTI RAMs at baseline (S147S/G and Q148Q/R), conferring resistance to raltegravir or elvitegravir; nonnucleoside reverse transcriptase inhibitor and protease inhibitor RAMs were observed in eight (25%) and three (9%) participants (Table 1). Mutations were not mutually exclusive.

**Table 1:** Baseline demographics and clinical characteristics B/FTC/TAF: bictegravir/emtricitabine/tenofovir alafenamide; ART: antiretroviral therapy; NRTI: nucleoside reverse transcriptase inhibitor; INSTI: integrase strand transfer inhibitor; NNRTI: nonnucleoside reverse transcriptase inhibitor; PI: protease inhibitor

Characteristic	Values (n = 40)
Male, n (%)	22 (55%)
Female, n (%)	18 (45%)
Black, n (%)	23 (57.5%)
Hispanic, n (%)	12 (30%)
Caucasian, n (%)	4 (10%)
Other, n (%)	1 (2.5%)
Median age (range)	58 years (27-81)
Mean weight (range)	87.7 kg (46-131)
Median time on B/FTC/TAF (range)	2.75 years (1-3.6)
Median time virologically suppressed prior to study entry (range)	7.5 years (0.6-14.2)
Median time since HIV diagnosis (range)	16.4 years (1.2-36)
Median number of ART regimens prior to enrollment (range)	5 (1-14)
Median baseline CD4 count (range)	681 cells/mm^3^ (372-1,044)
Median nadir CD4 count (range)	364 cell/mm^3^ (13-734)
Proviral DNA testing, n (%)	
NRTI	6 (19%)
M184I/V	6 (19%)
INSTI	2 (6%)
S147S/G	1 (3%)
Q148Q/R	1 (3%)
NNRTI	8 (25%)
K103K/N	6 (19%)
PI	3 (9%)
D30D/N	2 (6%)
N88S	1 (3%)
N88N/S	1 (3%)

Safety

No participants discontinued due to laboratory abnormalities or safety-related concerns. Median change from baseline in CD4+ cell count at week 96 was 38 cells/mm^3^. Thirty-four participants experienced one or more AEs while on treatment with DTG/3TC for a total of 81 events, none of which were Grade 3 or 4. The most common AEs included fatigue, headache, fever, viral illness, upper respiratory infection, abdominal pain, joint pain, muscle pain, and loose stool.   Five participants accounted for a total of six SAEs.  SAEs reported were endometrial cancer, symptomatic anemia, hiatal hernia, pulmonary nodule, acute pyelonephritis, and dizziness.  The glomerular filtration rate, creatinine, and fasting lipid profiles remained stable for over 96 weeks. Weight loss was observed at weeks 48 and 96, with an average decrease in weight of 3.3 kg from baseline (range: -24 to +6 kg) in post hoc analysis.

## Discussion

In this pilot trial, DTG/3TC was effective, with no PWH having an HIV-1 RNA ≥50 copies/mL at week 48 in the setting of unknown resistance. Furthermore, VS was maintained at the secondary endpoint, week 96. The presence of archived resistance, including M184I/V, did not affect viral suppression or lead to VF. Additionally, after two years of follow-up, no cases of treatment-emergent INSTI resistance were reported.

Several studies have demonstrated the efficacy of switching to DTG/3TC from a stable ART regimen through 48 weeks [12]. In the DYAD study, participants were randomized to either stay on B/FTC/TAF or switch to DTG/3TC; it was found that DTG/3TC was noninferior through 48 weeks [3]. The PASO DOBLE study also demonstrated noninferiority of DTG/3TC compared to B/FTC/TAF when switching from a stable regimen [13]. Our study, although smaller than the previously mentioned studies, provides efficacy data for DTG/3TC through 96 weeks.

Despite evidence supporting the efficacy of DTG/3TC, concerns about VF due to resistance remain. Some studies have reported efficacy data even in the absence of baseline resistance testing. In the DYAD study, for example, 44% of participants who were virologically suppressed on B/FTC/TAF and were switched to DTG/3TC had no genotype available at baseline, and DTG/3TC still performed well [3]. Of note, one case of M184M/I together with an INSTI secondary mutation (G140G/S, treatment-emergence unknown) was reported in the B/FTC/TAF arm [3]. In the SOLAR-3D trial, 100 participants, 50 with known MI84I/V and 50 without resistance testing at baseline, were evaluated for the efficacy of DTG/3TC at 48 weeks and found no cases of VF [4]. A meta-analysis investigating a stable switch to DTG/3TC in the presence of preexisting M184I/V mutations demonstrated that switching to DTG/3TC was effective in maintaining VS in patients with archived M184I/V mutations and found no cases of treatment-emergent INSTI resistance, even in the presence of historical or current M184V/I mutations, which is aligned to the results we report here [14].

Another important finding from this trial is the trend toward a decrease in weight from baseline. At two years, participants decreased their weight by 7.1 lbs (3.2 kg) without using other weight loss modalities. Since this was a single-arm study, we do not have a comparative arm; however, it is consistent with the PASO DOBLE study, which shows significantly less weight gain in suppressed PWH switching to DTG/3TC compared to switching to B/FTC/TAF. A similar finding was reported in the DRAGON study, which compared weight changes in treatment-naive Asian PWH who initiated either DTG/3TC or B/FTC/TAF [15]. The difference in the adjusted mean weight from baseline to week 48 in the DTG/3TC group was smaller than that observed in the B/FTC/TAF group; this finding is consistent with the results reported here [15]. In the previously mentioned DYAD study, there was no significant weight difference between those who switched to DTG/3TC compared to those who continued on B/FTC/TAF at 48 weeks. However, this could be due to the presence of a colocated weight loss health center.

The strengths of the SOUND study include its high percentage of Black (55%) and female (45%) participants, which adds to the diversity of the study population. Additionally, the study provides valuable data on the prevalence of RAMs and their impact on treatment outcomes. However, the study's limitations include its single-center, nonrandomized, single-arm design, which may limit the generalizability of the findings.

## Conclusions

In summary, the SOUND study supports the efficacy and safety of switching to DTG/3TC for people living with HIV-1 who are virologically suppressed on B/FTC/TAF with unknown resistance history. It also supports other studies' data, suggesting less weight gain with DTG/3TC compared to B/FTC/TAF. The study's findings are consistent with other real-world and interventional trials, providing additional evidence for the use of DTG/3TC in diverse populations.
